# Supplementary material for: Surface-mediated bacteriophage defense incurs fitness tradeoffs for interbacterial antagonism
Source: EMBO J. 2025 Mar 10;44(9):2473–500. doi: 10.1038/s44318-025-00406-3 (PMC12048535; doi:10.1038/s44318-025-00406-3)
Supplement: Supplementary file 1 — Appendix [file 44318_2025_406_MOESM1_ESM.pdf]

Appendix

Surface-mediated Bacteriophage Defense Incurs Fitness Tradeoffs for Interbacterial Antagonism

Chia-En Tsai[1,2,5], Feng-Qi Wang[2,5], Chih-Wen Yang[3], Ling-Li Yang[2], Thao V. Nguyen[2], Yung-Chih Chen[2], Po-Yin Chen[1,2], Ing-Shouh Hwang[3], See-Yeun Ting[1,2,4†]

[1] Molecular and Cell Biology, Taiwan International Graduate Program, Academia Sinica and National Defense Medical Center, Taipei, 11490, Taiwan

[2] Institute of Molecular Biology, Academia Sinica, Taipei 11529, Taiwan

[3] Institute of Physics, Academia Sinica, Taipei 115201, Taiwan

[4] Genome and Systems Biology Degree Program, National Taiwan University, Taipei 106319, Taiwan

[5] These authors contributed equally

[†]Correspondence: syting@gate.sinica.edu.tw

Table of contents:

Appendix Fig. S1:..........1-2
Appendix Fig. S2:..........3-4

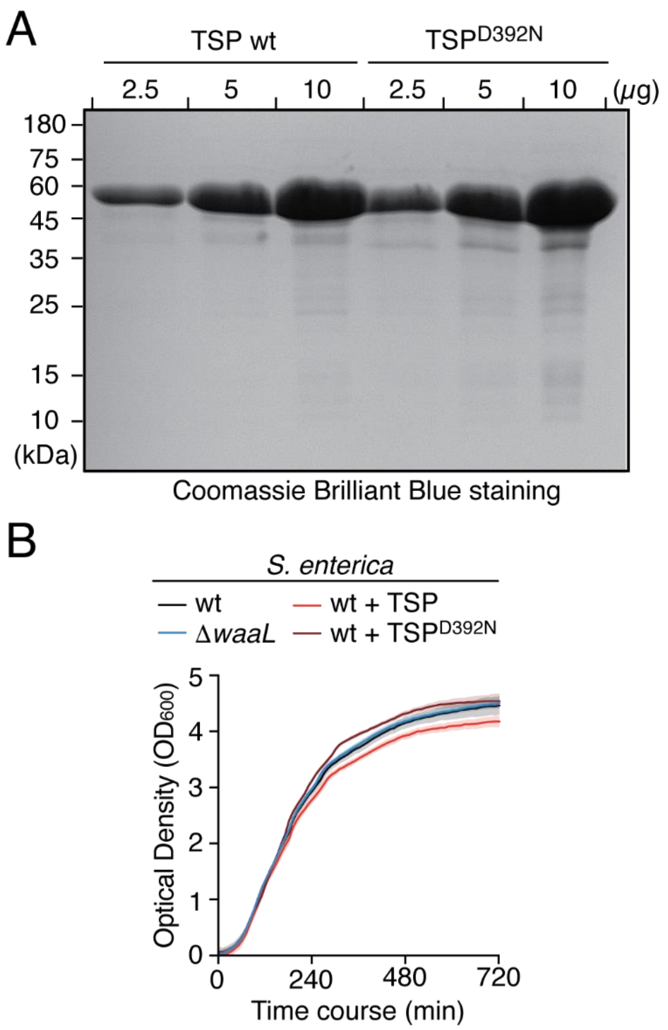
A
TSP wt
TSP^D392N
2.5
5
10
2.5
5
10
(μg)
180
75
60
45
35
25
15
10
(kDa)
Coomassie Brilliant Blue staining
B
S. enterica
wt
ΔwaaL
wt + TSP
wt + TSP^D392N
Optical Density (OD600)
Time course (min)
0
1
2
3
4
5
0
240
480
720

Appendix Fig. S1. TSP-treated *S. enterica* shows no significant growth defect. (A) Coomassie-stained 12.5% SDS-PAGE analysis of the purified TSP (left) and the D392N catalytic mutant (right). **(B)** Growth curves of wild-type *S. enterica* incubated with 10 nM of purified TSP (wild-type or D392N mutant) at 37 °C. Mock-treated wild-type *S. enterica* and the Δ*waaL* mutant are shown as controls.

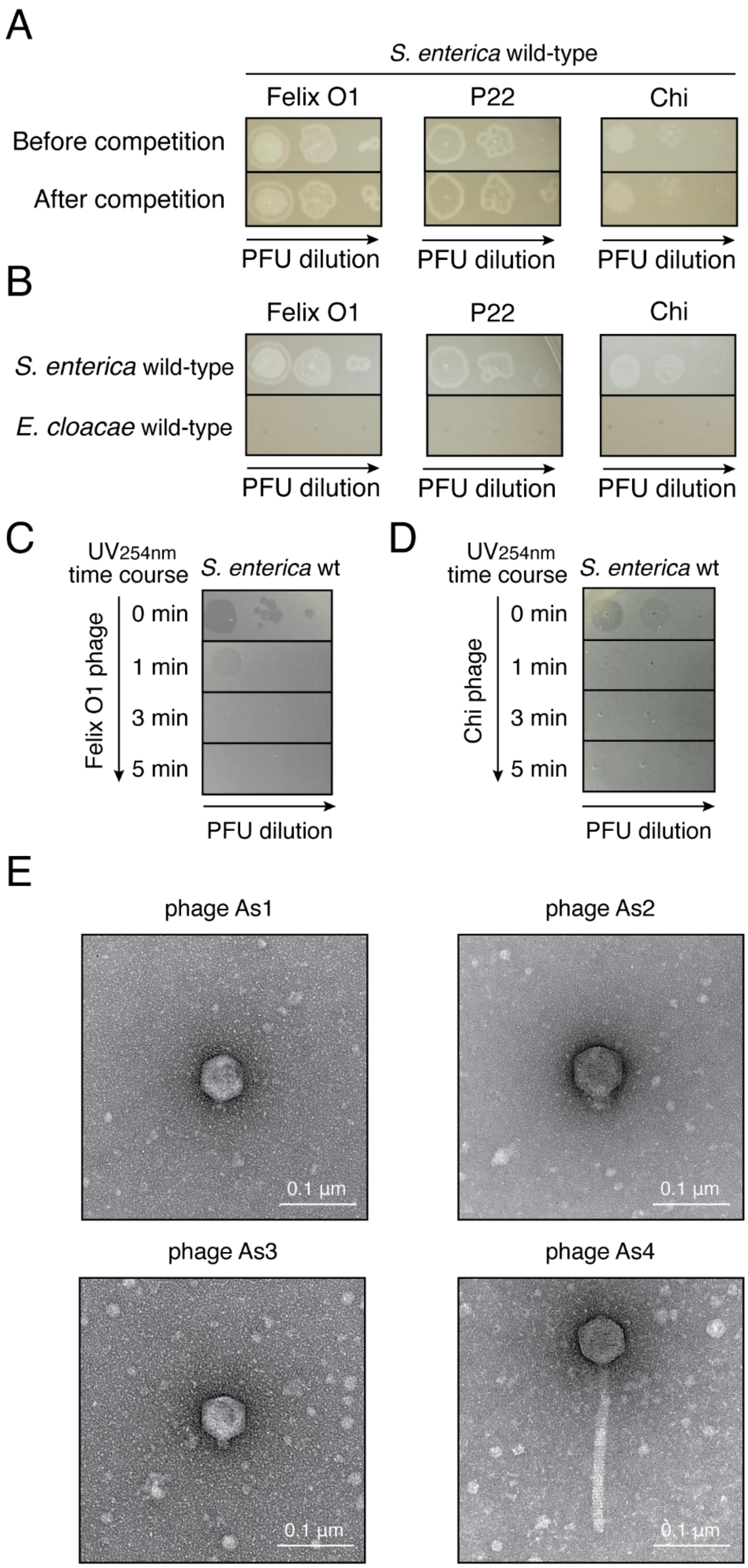
A
S. enterica wild-type
Felix O1
P22
Chi
Before competition
After competition
PFU dilution
PFU dilution
PFU dilution
B
Felix O1
P22
Chi
S. enterica wild-type
E. cloacae wild-type
PFU dilution
PFU dilution
PFU dilution
C
UV254nm time course
S. enterica wt
Felix O1 phage
0 min
1 min
3 min
5 min
PFU dilution
D
UV254nm time course
S. enterica wt
Chi phage
0 min
1 min
3 min
5 min
PFU dilution
E
phage As1
phage As2
phage As3
phage As4
0.1 µm
0.1 µm
0.1 µm
0.1 µm

Appendix Fig. S2. Supporting evidence that phage P22 sensitizes *S. enterica* to T6SS-mediated attacks. (A) Ten-fold serial dilution spot assay of phages Felix O1 (left), P22 (middle), and Chi (right) on wild-type *S. enterica* before and after the competition assay (**Fig 5B**). **(B)** Ten-fold serial dilution spot assay of phages Felix O1 (left), P22 (middle), and Chi (right) on wild-type *S. enterica* and *E. cloacae.* **(C, D)** Ten-fold serial dilution spot assay of UV-inactivated Felix O1 phage **(C)** or Chi phage **(D)** on wild-type *S. enterica*. The images are representative of triplicate experiments. **(E)** TEM images of isolated phages used in the study: As1 (top left), As2 (top right), As3 (bottom left), and As4 (bottom right). Scale bar = 0.1 μm.
