# Supplementary material for: Surface-mediated bacteriophage defense incurs fitness tradeoffs for interbacterial antagonism
Source: EMBO J. 2025 Mar 10;44(9):2473–500. doi: 10.1038/s44318-025-00406-3 (PMC12048535; doi:10.1038/s44318-025-00406-3)
Supplement: Supplementary file 2 — Table EV1 [file 44318_2025_406_MOESM2_ESM.docx]

**Table EV1. List of bacterial and bacteriophage strains used in this study.**

| **Strain** | **Source** | **Identifier** |
| --- | --- | --- |
| *Escherichia coli* DH5α | YB Biotech | Cat# XR-LYE678-80 |
| *Escherichia coli* PIR1 | Gift from Dr. Hsin-Hung David Chou | N/A |
| *Escherichia coli* BL21 pRIL (DE3) | Gift from Dr. Hung-Ta Chen | N/A |
| *Escherichia coli* S17-1 λ pir | Lab collection | N/A |
| *Enterobacter cloacae* | American Type Culture Collection | ATCC 13047 |
| *Enterobacter cloacae* ∆*tssM* (∆*ECL_01536*) | Whitney *et al.*, 2014 | N/A |
| *Enterobacter cloacae* ∆*cdiA* | Beck *et al.*, 2015 | N/A |
| *Salmonella enterica* serovar Typhimurium | American Type Culture Collection | ATCC 14028 |
| *Salmonella enterica* serovar Typhimurium Tn7::Chl | This paper | N/A |
| *Salmonella enterica* serovar Typhimurium LT2/(P22)+ | Bioresource Collection and Research Center | BCRC 12459 |
| *Salmonella enterica* bacteriophage Felix O1 | Leibniz Institute DSMZ | DSM 18524 |
| *Salmonella enterica* bacteriophage Chi | American Type Culture Collection | ATCC 9842-B1 |
| *Salmonella enterica* bacteriophage As1 | This paper | N/A |
| *Salmonella enterica* bacteriophage As2 | This paper | N/A |
| *Salmonella enterica* bacteriophage As3 | This paper | N/A |
| *Salmonella enterica* bacteriophage As4 | This paper | N/A |
| *Salmonella enterica* serovar Typhimurium Tn7::Chl ∆*waaL* | This paper | N/A |
| *Salmonella enterica* serovar Typhimurium Tn7::Chl ∆*waaJ* | This paper | N/A |
| *Salmonella enterica* serovar Typhimurium Tn7::Chl ∆*waaO* | This paper | N/A |
| *Salmonella enterica* serovar Typhimurium Tn7::Chl ∆*waaG* | This paper | N/A |
| *Salmonella enterica* serovar Typhimurium Tn7::Chl ∆*waaK* | This paper | N/A |
| *Salmonella enterica* serovar Typhimurium Tn7::Chl ∆*waaB* | This paper | N/A |
| *Salmonella enterica* serovar Typhimurium Tn7::Chl ∆*waaQ* | This paper | N/A |
| *Salmonella enterica* serovar Typhimurium Tn7::Chl ∆*waaY* | This paper | N/A |
| *Salmonella enterica* serovar Typhimurium Tn7::Chl ∆*wzzB* | This paper | N/A |
| *Salmonella enterica* serovar Typhimurium Tn7::Chl ∆*fepE* | This paper | N/A |
| Enterohemorrhagic *Escherichia coli* O157:H7 EDL933 | American Type Culture Collection | ATCC-43895 |
| Enterohemorrhagic *Escherichia coli* O157:H7 EDL933 Tn7::Chl | This paper | N/A |
| Enterohemorrhagic *Escherichia coli* O157:H7 EDL933 Tn7::Chl ∆*waaO* | This paper | N/A |
| *Citrobacter rodentium* DBS100 | American Type Culture Collection | ATCC-51459 |
| *Citrobacter rodentium* DBS100 ∆*waaO* | This paper | N/A |
| *Burkholderia thailandensis* E264 | American Type Culture Collection | ATCC-700388 |
| *Burkholderia thailandensis* E264 ∆*tssM* (∆*BTH_I2954*) | Schwarz *et al.,* 2010  LeRoux *et al.*, 2015 | N/A |
| Felix O1-resistant *Salmonella enterica* serovar Typhimurium-R1 | This paper | N/A |
| Felix O1-resistant *Salmonella enterica* serovar Typhimurium-R2 | This paper | N/A |
| Felix O1-resistant *Salmonella enterica* serovar Typhimurium-R3 | This paper | N/A |
| Felix O1-resistant *Salmonella enterica* serovar Typhimurium-R4 | This paper | N/A |
| Felix O1-resistant *Salmonella enterica* serovar Typhimurium-R5 | This paper | N/A |
| Felix O1-resistant *Salmonella enterica* serovar Typhimurium-R6 | This paper | N/A |
| Felix O1-resistant *Salmonella enterica* serovar Typhimurium-R7 | This paper | N/A |
| Felix O1-resistant *Salmonella enterica* serovar Typhimurium-R8 | This paper | N/A |
| Felix O1-resistant *Salmonella enterica* serovar Typhimurium-R9 | This paper | N/A |
| Felix O1-resistant *Salmonella enterica* serovar Typhimurium-R10 | This paper | N/A |
| Felix O1-resistant *Salmonella enterica* serovar Typhimurium-R11 | This paper | N/A |
| Felix O1-resistant *Salmonella enterica* serovar Typhimurium-R12 | This paper | N/A |
| Felix O1-resistant *Salmonella enterica* serovar Typhimurium-R13 | This paper | N/A |
| Felix O1-resistant *Salmonella enterica* serovar Typhimurium-R14 | This paper | N/A |
| Felix O1-resistant *Salmonella enterica* serovar Typhimurium-R15 | This paper | N/A |
| Felix O1-resistant *Salmonella enterica* serovar Typhimurium-R16 | This paper | N/A |
| Felix O1-resistant *Salmonella enterica* serovar Typhimurium-R17 | This paper | N/A |
| Felix O1-resistant *Salmonella enterica* serovar Typhimurium-R18 | This paper | N/A |
| P22-resistant *Salmonella enterica* serovar Typhimurium-R1 | This paper | N/A |
| P22-resistant *Salmonella enterica* serovar Typhimurium-R2 | This paper | N/A |
| P22-resistant *Salmonella enterica* serovar Typhimurium-R3 | This paper | N/A |
| P22-resistant *Salmonella enterica* serovar Typhimurium-R4 | This paper | N/A |
| P22-resistant *Salmonella enterica* serovar Typhimurium-R5 | This paper | N/A |
| P22-resistant *Salmonella enterica* serovar Typhimurium-R6 | This paper | N/A |
| P22-resistant *Salmonella enterica* serovar Typhimurium-R7 | This paper | N/A |
| P22-resistant *Salmonella enterica* serovar Typhimurium-R8 | This paper | N/A |
| P22-resistant *Salmonella enterica* serovar Typhimurium-R9 | This paper | N/A |
| P22-resistant *Salmonella enterica* serovar Typhimurium-R10 | This paper | N/A |
| P22-resistant *Salmonella enterica* serovar Typhimurium-R11 | This paper | N/A |
| P22-resistant *Salmonella enterica* serovar Typhimurium-R12 | This paper | N/A |
| P22-resistant *Salmonella enterica* serovar Typhimurium-R13 | This paper | N/A |
| P22-resistant *Salmonella enterica* serovar Typhimurium-R14 | This paper | N/A |
| P22-resistant *Salmonella enterica* serovar Typhimurium-R15 | This paper | N/A |
| P22-resistant *Salmonella enterica* serovar Typhimurium-R16 | This paper | N/A |
| P22-resistant *Salmonella enterica* serovar Typhimurium-R17 | This paper | N/A |
| P22-resistant *Salmonella enterica* serovar Typhimurium-R18 | This paper | N/A |
| Chi-resistant *Salmonella enterica* serovar Typhimurium-R1 | This paper | N/A |
| Chi-resistant *Salmonella enterica* serovar Typhimurium-R2 | This paper | N/A |
| Chi-resistant *Salmonella enterica* serovar Typhimurium-R3 | This paper | N/A |
| Chi-resistant *Salmonella enterica* serovar Typhimurium-R4 | This paper | N/A |
| Chi-resistant *Salmonella enterica* serovar Typhimurium-R5 | This paper | N/A |
| Chi-resistant *Salmonella enterica* serovar Typhimurium-R6 | This paper | N/A |
| Chi-resistant *Salmonella enterica* serovar Typhimurium-R7 | This paper | N/A |
| Chi-resistant *Salmonella enterica* serovar Typhimurium-R8 | This paper | N/A |
| Chi-resistant *Salmonella enterica* serovar Typhimurium-R9 | This paper | N/A |
