# Supplementary material for: Surface-mediated bacteriophage defense incurs fitness tradeoffs for interbacterial antagonism
Source: EMBO J. 2025 Mar 10;44(9):2473–500. doi: 10.1038/s44318-025-00406-3 (PMC12048535; doi:10.1038/s44318-025-00406-3)
Supplement: Supplementary file 3 — Table EV2 [file 44318_2025_406_MOESM3_ESM.docx]

**Table EV2. List of primers used in this study.**

| **Primer** | **Sequences (5'-3')** | **Identifier** |
| --- | --- | --- |
| **Bacteria strain construction** |  |  |
| Sent_∆waaL_F1 | CATGAATTCCCGGGAGAGCTCATCAAAGCGGAACGCACAG | Mission Biotech |
| Sent_∆waaL_R1 | TTTATCTATTGGTTAGCATCTTTTCTCCACAATAG | Mission Biotech |
| Sent_∆waaL_F2 | GATGCTAACCAATAGATAAAAAAACGCGC | Mission Biotech |
| Sent_∆waaL_R2 | CGACGGATCCCAAGCTTCTTCTAGAACCAGAACTTCCTGATAATG | Mission Biotech |
| Sent_∆waaL_seq_F | TGATTCCCGTCGCTATTCGC | Mission Biotech |
| Sent_∆waaL_seq_R | AGATCAGAGAGCGCAATCCG | Mission Biotech |
| Sent_∆waaJ_F1 | CATGAATTCCCGGGAGAGCTCGTACGCTACGCAGATTGTTG | Mission Biotech |
| Sent_∆waaJ_R1 | ATTATTTGTGTGAATCCATTACATCACCTATG | Mission Biotech |
| Sent_∆waaJ_F2 | AATGGATTCACACAAATAATTCCCCTCC | Mission Biotech |
| Sent_∆waaJ_R2 | CGACGGATCCCAAGCTTCTTCTAGACCTTACCCTTAATACGAC | Mission Biotech |
| Sent_∆waaJ_seq_F | TGAAGACCAGCAGCGATTTG | Mission Biotech |
| Sent_∆waaJ_seq_R | ACGTGCTACATTGATCAGCG | Mission Biotech |
| Sent_∆waaO_F1 | CATGAATTCCCGGGAGAGCTCGTGATATGTATTGATGTCATCTC | Mission Biotech |
| Sent_∆waaO_R1 | TTTATTCAAGTCTGCTCATAGTGATCAC | Mission Biotech |
| Sent_∆waaO_F2 | TATGAGCAGACTTGAATAAAACCCATAGGTG | Mission Biotech |
| Sent_∆waaO_R2 | CGACGGATCCCAAGCTTCTTCTAGAGTGACTTATCTTTCAACTCAC | Mission Biotech |
| Sent_∆waaO_seq_F | CTTCGTCGAGCTAAGCATG | Mission Biotech |
| Sent_∆waaO_seq_R | TGTAGCGCCCGTATAATG | Mission Biotech |
| Sent_∆waaG_F1 | CATGAATTCCCGGGAGAGCTCAATCTCACCGATCAGTGG | Mission Biotech |
| Sent_∆waaG_R1 | CTCAACCATCAACTCTCATAGCAGGCTG | Mission Biotech |
| Sent_∆waaG_F2 | TATGAGAGTTGATGGTTGAGCTGAAAGC | Mission Biotech |
| Sent_∆waaG_R2 | CGACGGATCCCAAGCTTCTTCTAGACACTCATAAATTACTCACTGAG | Mission Biotech |
| Sent_∆waaG_seq_F | AGATCAAAAACGCGCTATCG | Mission Biotech |
| Sent_∆waaG_seq_R | GCAATGTCCAGTCTTCTTCC | Mission Biotech |
| Sent_∆waaK_F1 | CATGAATTCCCGGGAGAGCTCAAGATGTCATTGCAGTCAATG | Mission Biotech |
| Sent_∆waaK_R1 | ATCACTTATCTTTAATCATAATAGCTATAATCCAGAATAG | Mission Biotech |
| Sent_∆waaK_F2 | TATGATTAAAGATAAGTGATTGAGTCCTG | Mission Biotech |
| Sent_∆waaK_R2 | CGACGGATCCCAAGCTTCTTCTAGACATCGACATATGTCCGATTC | Mission Biotech |
| Sent_∆waaK_seq_F | GGTCCTACGTCCAGAAAGAC | Mission Biotech |
| Sent_∆waaK_seq_R | TTTAGGACTTCGCTGCCTTG | Mission Biotech |
| Sent_∆waaB_F1 | CATGAATTCCCGGGAGAGCTCGAAGACTACTGTGCTGAC | Mission Biotech |
| Sent_∆waaB_R1 | ATCACTTTTGTATTTTCATATATACCCCAGAG | Mission Biotech |
| Sent_∆waaB_F2 | TATGAAAATACAAAAGTGATCACTATGAGC | Mission Biotech |
| Sent_∆waaB_R2 | CGACGGATCCCAAGCTTCTTCTAGATACAGTCTCAGCATCAAC | Mission Biotech |
| Sent_∆waaB_seq_F | TGGGCGTTGATACGATGCAC | Mission Biotech |
| Sent_∆waaB_seq_R | ACTATGCCAGGGCTTCGTTG | Mission Biotech |
| Sent_∆waaQ_F1 | CATGAATTCCCGGGAGAGCTCGTGCAGCGCAGCAATCAG | Mission Biotech |
| Sent_∆waaQ_R1 | CTCATAGCAGCTTTTCCACAAGCGACTCTTTG | Mission Biotech |
| Sent_∆waaQ_F2 | TGTGGAAAAGCTGCTATGAGAGTTGCCTTTTG | Mission Biotech |
| Sent_∆waaQ_R2 | CGACGGATCCCAAGCTTCTTCTAGACCATTAACTCCGCGATATC | Mission Biotech |
| Sent_∆waaQ_seq_F | GCGCATTAGCGATAACCAGG | Mission Biotech |
| Sent_∆waaQ_seq_R | CCTGATAGGCTGGATGCAG | Mission Biotech |
| Sent_∆waaY_F1 | for making Salmonella 14028 ChlR waaY/rfaY (EJJ31_RS00655) deletion with pRE118 | Mission Biotech |
| Sent_∆waaY_R1 | ATCAGCGTTTAATAATCATAATGGAGATTTAGGG | Mission Biotech |
| Sent_∆waaY_F2 | TATGATTATTAAACGCTGATCAATGTAG | Mission Biotech |
| Sent_∆waaY_R2 | CGACGGATCCCAAGCTTCTTCTAGAATTCGTCATAGAAACGTG | Mission Biotech |
| Sent_∆waaY_seq_F | GTTTAGCCGTACAGCATCAC | Mission Biotech |
| Sent_∆waaY_seq_R | TAAGCTCAGATGGCATAGGG | Mission Biotech |
| Sent_∆wzzB_F1 | CATGAATTCCCGGGAGAGCTCACTCCAGGAAGGCGCTATTAAAC | Mission Biotech |
| Sent_∆wzzB_R1 | CTTACAAGGCCACTGTCATAGATACCCTAACTAAAAAAAGGATG | Mission Biotech |
| Sent_∆wzzB_F2 | TATGACAGTGGCCTTGTAAGCTTCTTTGCCGGATG | Mission Biotech |
| Sent_∆wzzB_R2 | CGACGGATCCCAAGCTTCTTCTAGATGTCGACGGCGCGC | Mission Biotech |
| Sent_∆wzzB_seq_F | CGTGATTGGGGAACAGTCTG | Mission Biotech |
| Sent_∆wzzB_seq_R | CTTGAGGCATTCCGTGATGC | Mission Biotech |
| Sent_∆fepE_F1 | CATGAATTCCCGGGAGAGCTCCTATACCGTCGCAAAAAACC | Mission Biotech |
| Sent_∆fepE_R1 | CATGCCATCTTTAGTCTGATTTACCCGAAAAGC | Mission Biotech |
| Sent_∆fepE_F2 | ATCAGACTAAAGATGGCATGCCGTATGAC | Mission Biotech |
| Sent_∆fepE_R2 | CGACGGATCCCAAGCTTCTTCTAGAAGGTATGTGAACATCATC | Mission Biotech |
| Sent_∆fepE_seq_F | CCTATGCAAACCGCAACCACCC | Mission Biotech |
| Sent_∆fepE_seq_R | CCGAATCCGTAGCCCGTTTGC | Mission Biotech |
| Sent_WzzB_F | ACAATTTCAGAATTCGAGCTCATGACAGTGGATAGTAATACGTC | Mission Biotech |
| Sent_WzzB_R | TCATTTCAATATCTGTATATCTAGATTACAAGGCTTTTGGCTTATAGC | Mission Biotech |
| Sent_FepE_F | ACAATTTCAGAATTCGAGCTCATGCCATCTCTTAATGTAAAACAAG | Mission Biotech |
| Sent_FepE_R | TCATTTCAATATCTGTATATCTAGATCAGACTAACCGTTCATCTATC | Mission Biotech |
| Ecoli_∆waaO_F1 | CATGAATTCCCGGGAGAGCTCCGTTATGGCGCGGTAAAG | Mission Biotech |
| Ecoli_∆waaO_R1 | TTTAGAAGCATTGAGACATATTTATTCTGTATCCAAAATTTTG | Mission Biotech |
| Ecoli_∆waaO_F2 | TATGTCTCAATGCTTCTAAAGATTTAAGGTTAAAATAATTC | Mission Biotech |
| Ecoli_∆waaO_R2 | CGACGGATCCCAAGCTTCTTCTAGAGCGCTTGTCCAGTTCAATTATATC | Mission Biotech |
| Ecoli_∆waaO_seq_F | GATGGTTGAACTTAAAGAGCCG | Mission Biotech |
| Ecoli_∆waaO_seq_R | CCTGGTAGTTCTCATCAATACC | Mission Biotech |
| Crod_∆waaO_F1 | CATGAATTCCCGGGAGAGCTCCGTTATGGCGCGGTAAAG | Mission Biotech |
| Crod_∆waaO_R1 | TTTAGAAGCATTGAGACATATTTATTCTGTATCCAAAATTTTG | Mission Biotech |
| Crod_∆waaO_F2 | TATGTCTCAATGCTTCTAAAGATTTAAGGTTAAAATAATTC | Mission Biotech |
| Crod_∆waaO_R2 | CGACGGATCCCAAGCTTCTTCTAGAGCGCTTGTCCAGTTCAATTATATC | Mission Biotech |
| Crod_∆waaO_seq_F | ATTAATGCATTTAGCCAGTGGC | Mission Biotech |
| Crod_∆waaO_seq_R | TGTATCTTCTGAAATTATGCGC | Mission Biotech |
| **Plasmid construction** |  |  |
| pETDuet_TSP_F | ACCATCATCACCACAGCCAGGATCCAGATCAATATTCAATAGAAGC | Mission Biotech |
| pETDuet_TSP_R | GTTCGACTTAAGCATTATGCGGCCGCTAAAGTGTTGCCAAGGTTAATCTAACTAATTC | Mission Biotech |
| pETDuet_TSP_D392N_F | CCCCGTATGGAATGGATTCGATTTAGGTGCTGAC | Mission Biotech |
| pETDuet_TSP_D392N_R | CGAATCCATTCCATACGGGGTAAATAACGACC | Mission Biotech |
| pETDuet_Seq_F | ATGCGTCCGGCGTAGA | Mission Biotech |
| pETDuet_Seq_R | GATTATGCGGCCGTGTACAA | Mission Biotech |
| pRE118_seq_F | GGTTGAGAAGCGGTGTAAGTG | Mission Biotech |
| pRE118_seq_R | GCAGGTCGATCCCAAGCTTC | Mission Biotech |
| pPSV39_seq_F | GGATTACGTGGCCTGTAGAC | Mission Biotech |
| pPSV39_seq_R | CCCGTTTAGAGGCCCC | Mission Biotech |
| **Transposon Insertion mapping** |  |  |
| EzTn5_s | GATCCTCTAGAGTCGACCTGCAGGCATGCA | Integrated DNA Technologies, Inc. |
| Tn5_F | TCGTCGGCAGCGTCAGATGTGTATAAGAGACAG | Integrated DNA Technologies, Inc. |
| Tn5_R | GTCTCGTGGGCTCGGAGATGTGTATAAGAGACAGGGGGGGGGGGGGGGG | Integrated DNA Technologies, Inc. |
