# Supplementary figures and images for: Surface-mediated bacteriophage defense incurs fitness tradeoffs for interbacterial antagonism
Source: EMBO J. 2025 Mar 10;44(9):2473–500. doi: 10.1038/s44318-025-00406-3 (PMC12048535; doi:10.1038/s44318-025-00406-3)

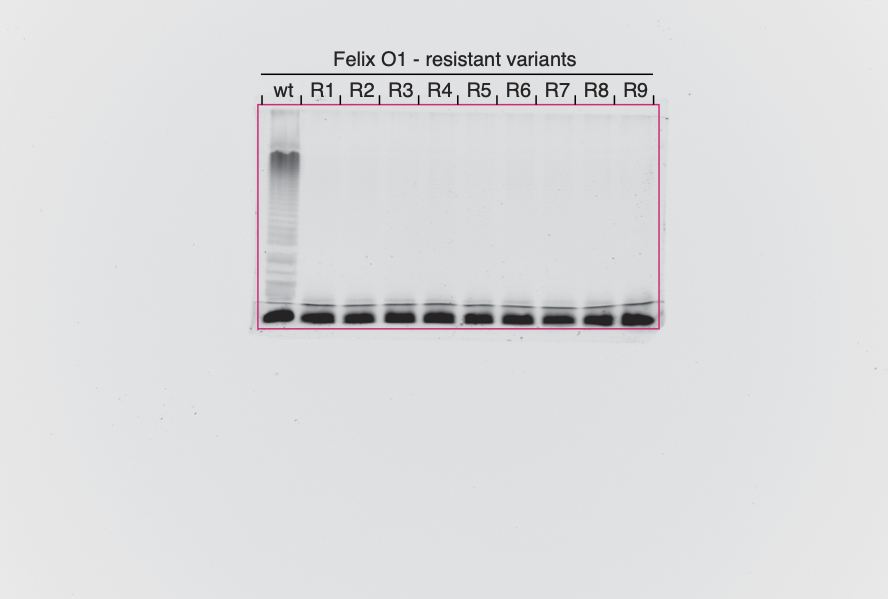

Supplement: Supplementary file 6 — Source data Fig. 2 [file 44318_2025_406_MOESM6_ESM.zip › Source data_Fig. 2/2A/Fig. 2A_LPS SDS_Felix O1.tiff]

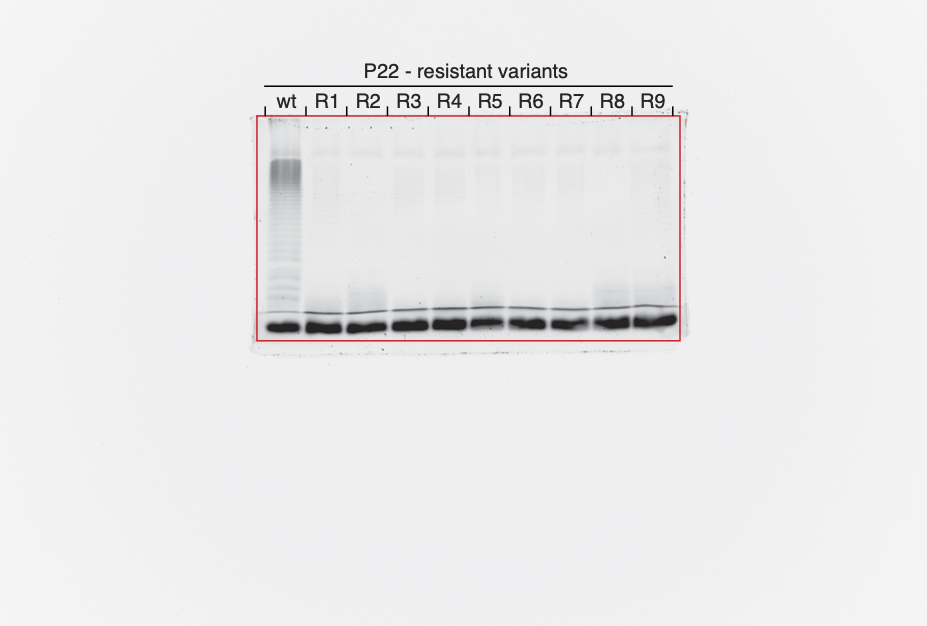

Supplement: Supplementary file 6 — Source data Fig. 2 [file 44318_2025_406_MOESM6_ESM.zip › Source data_Fig. 2/2B/Fig. 2B_LPS SDS_P22.tiff]

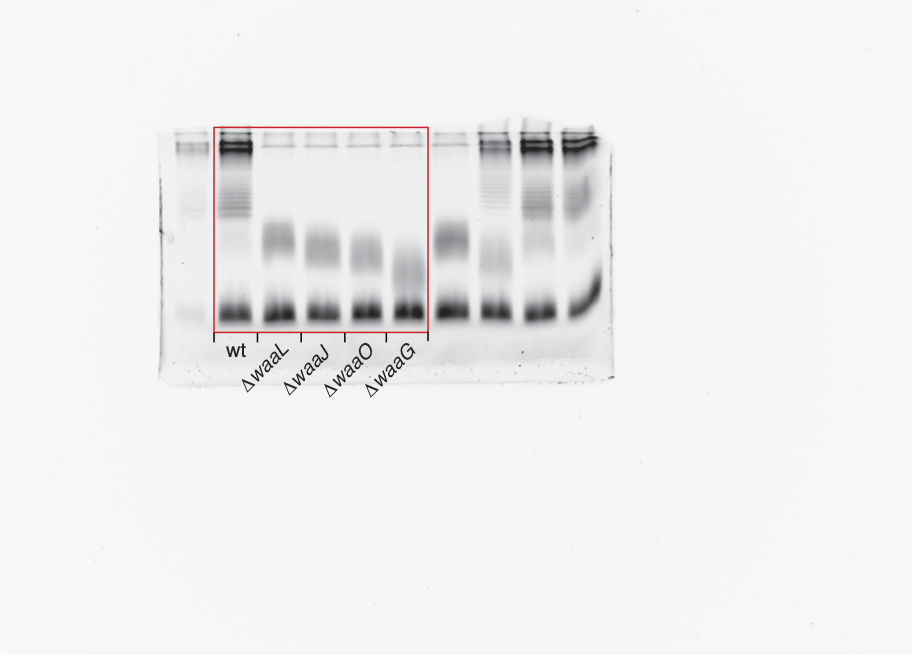

Supplement: Supplementary file 7 — Source data Fig. 3 [file 44318_2025_406_MOESM7_ESM.zip › Source data_Fig. 3/3C/Fig. 3C-LPS SDS_main.tiff]

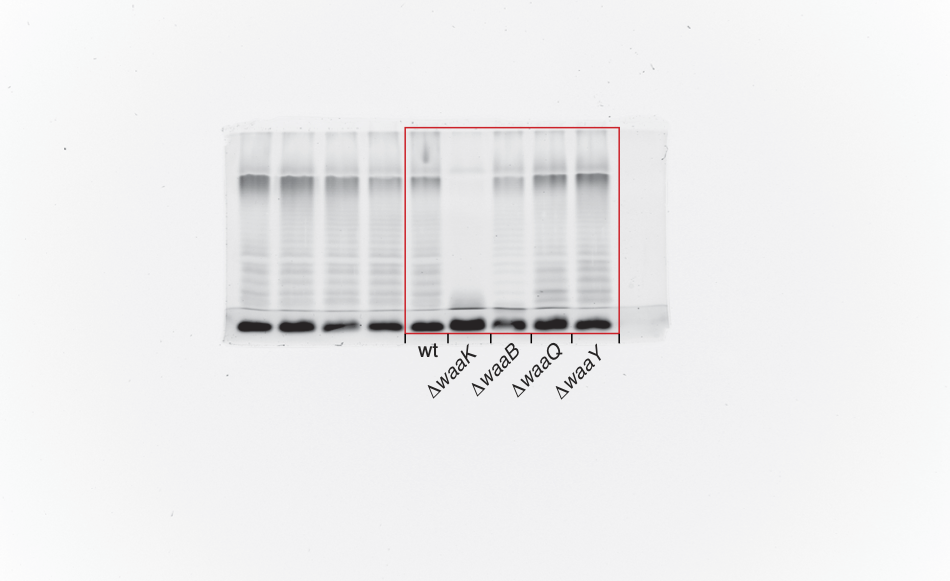

Supplement: Supplementary file 7 — Source data Fig. 3 [file 44318_2025_406_MOESM7_ESM.zip › Source data_Fig. 3/3E/Fig. 3E-LPS SDS_side.tiff]

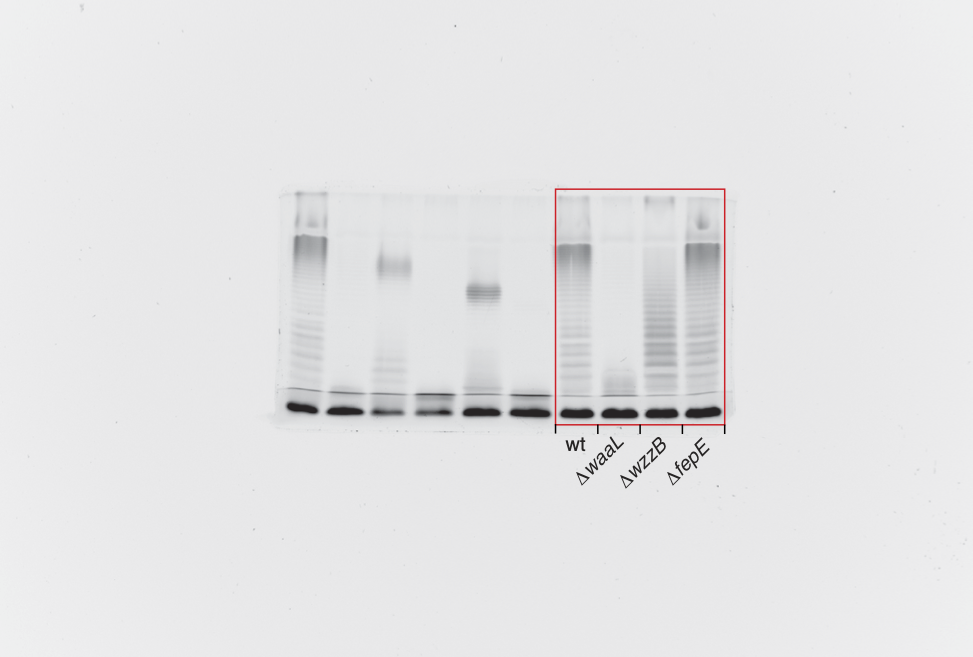

Supplement: Supplementary file 7 — Source data Fig. 3 [file 44318_2025_406_MOESM7_ESM.zip › Source data_Fig. 3/3F/Fig. 3F-LPS SDS_O-Ag.tiff]

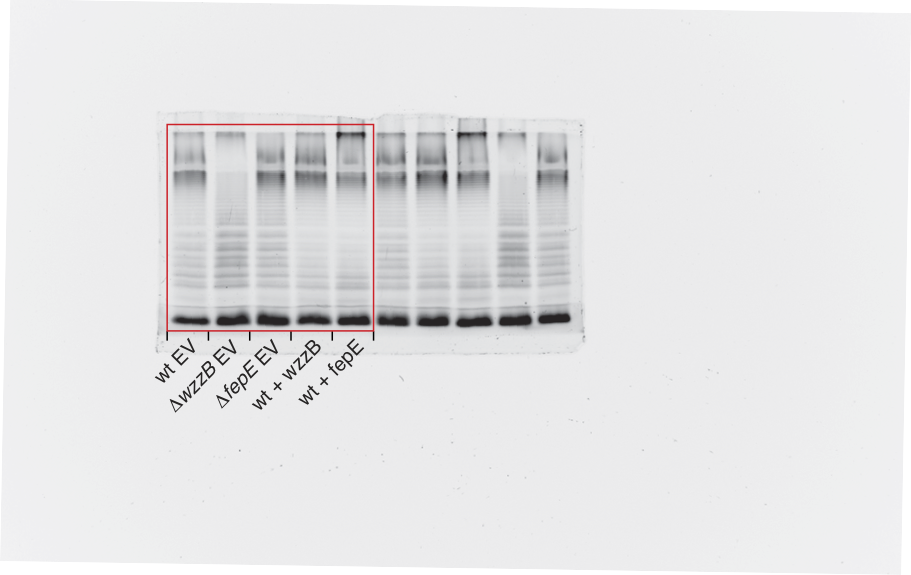

Supplement: Supplementary file 7 — Source data Fig. 3 [file 44318_2025_406_MOESM7_ESM.zip › Source data_Fig. 3/3H/Fig. 3H-LPS SDS_O-Ag o_e.tiff]

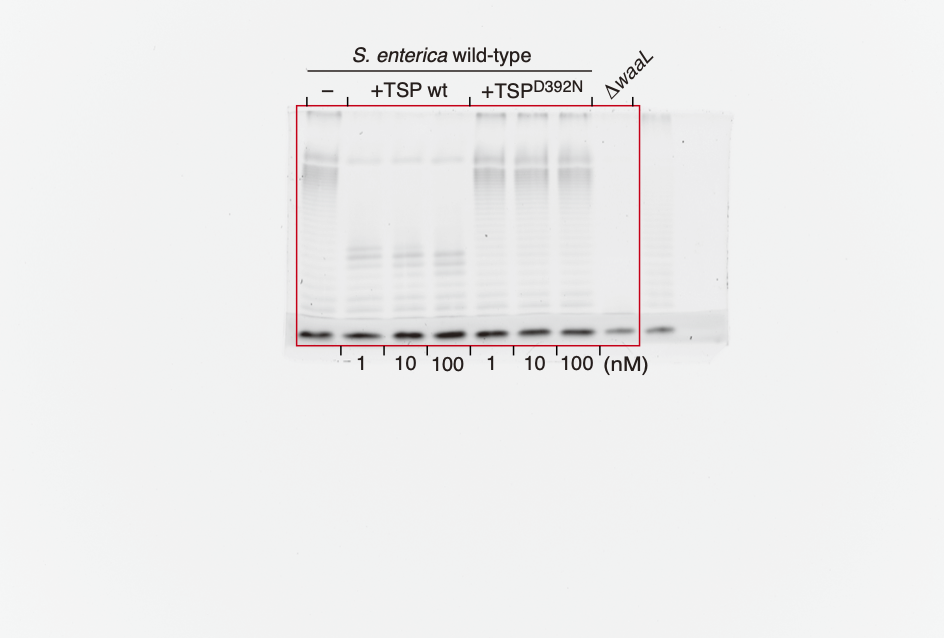

Supplement: Supplementary file 8 — Source data Fig. 4 [file 44318_2025_406_MOESM8_ESM.zip › Source data_Fig. 4/4D/Fig. 4D-LPS SDS_TSP.tiff]

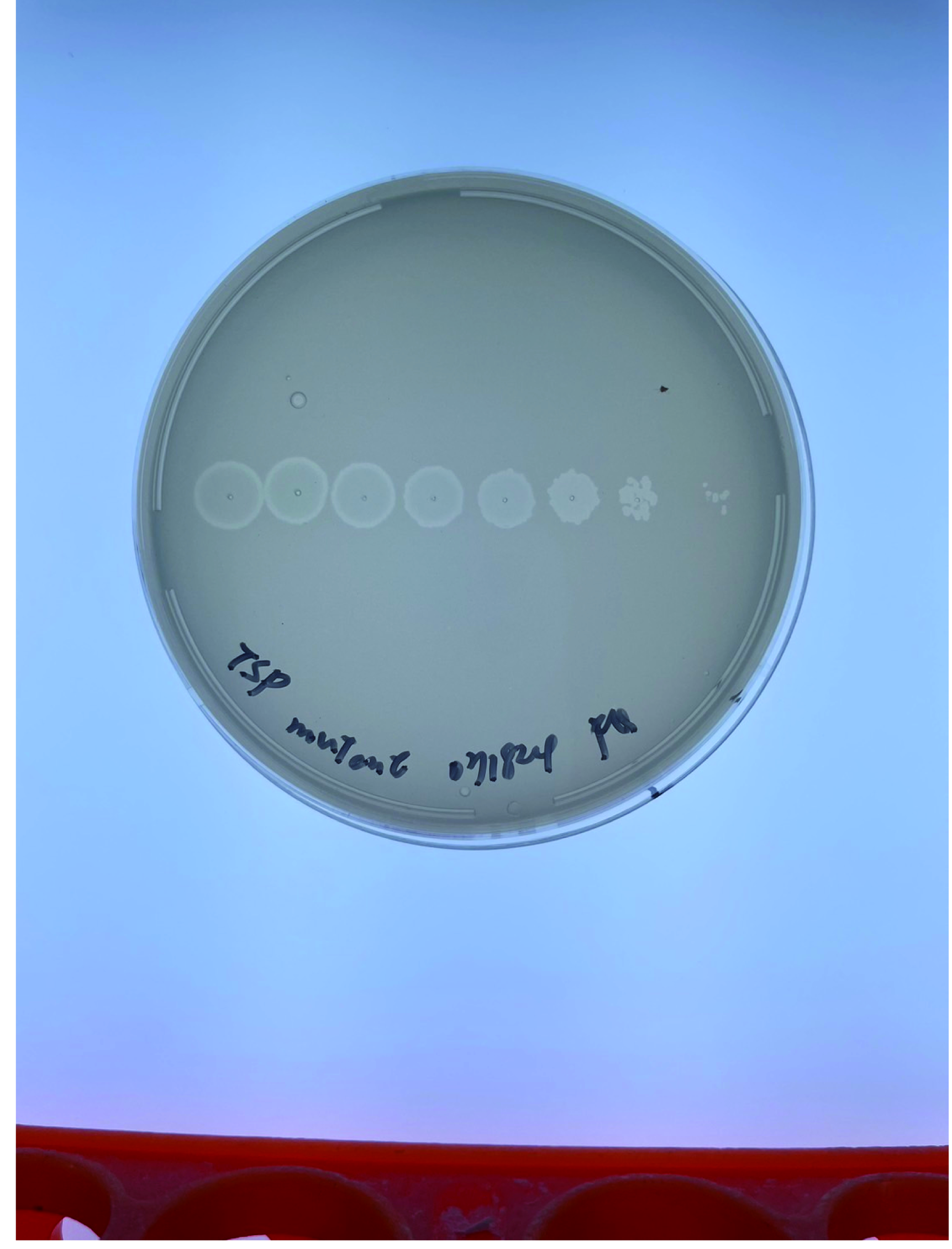

Supplement: Supplementary file 8 — Source data Fig. 4 [file 44318_2025_406_MOESM8_ESM.zip › Source data_Fig. 4/4E/TSP catalytic mutant.tif]

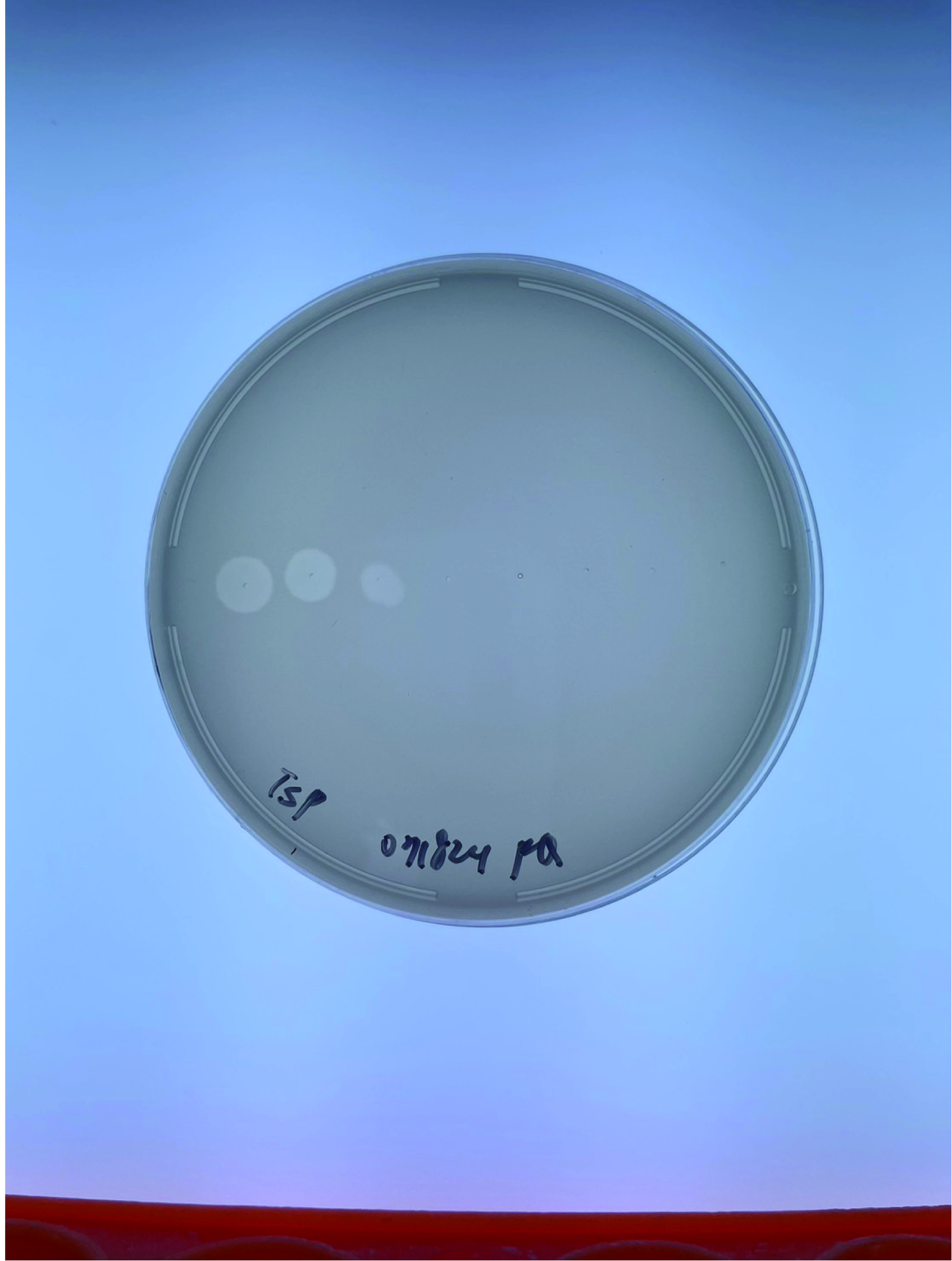

Supplement: Supplementary file 8 — Source data Fig. 4 [file 44318_2025_406_MOESM8_ESM.zip › Source data_Fig. 4/4E/TSP.tif]

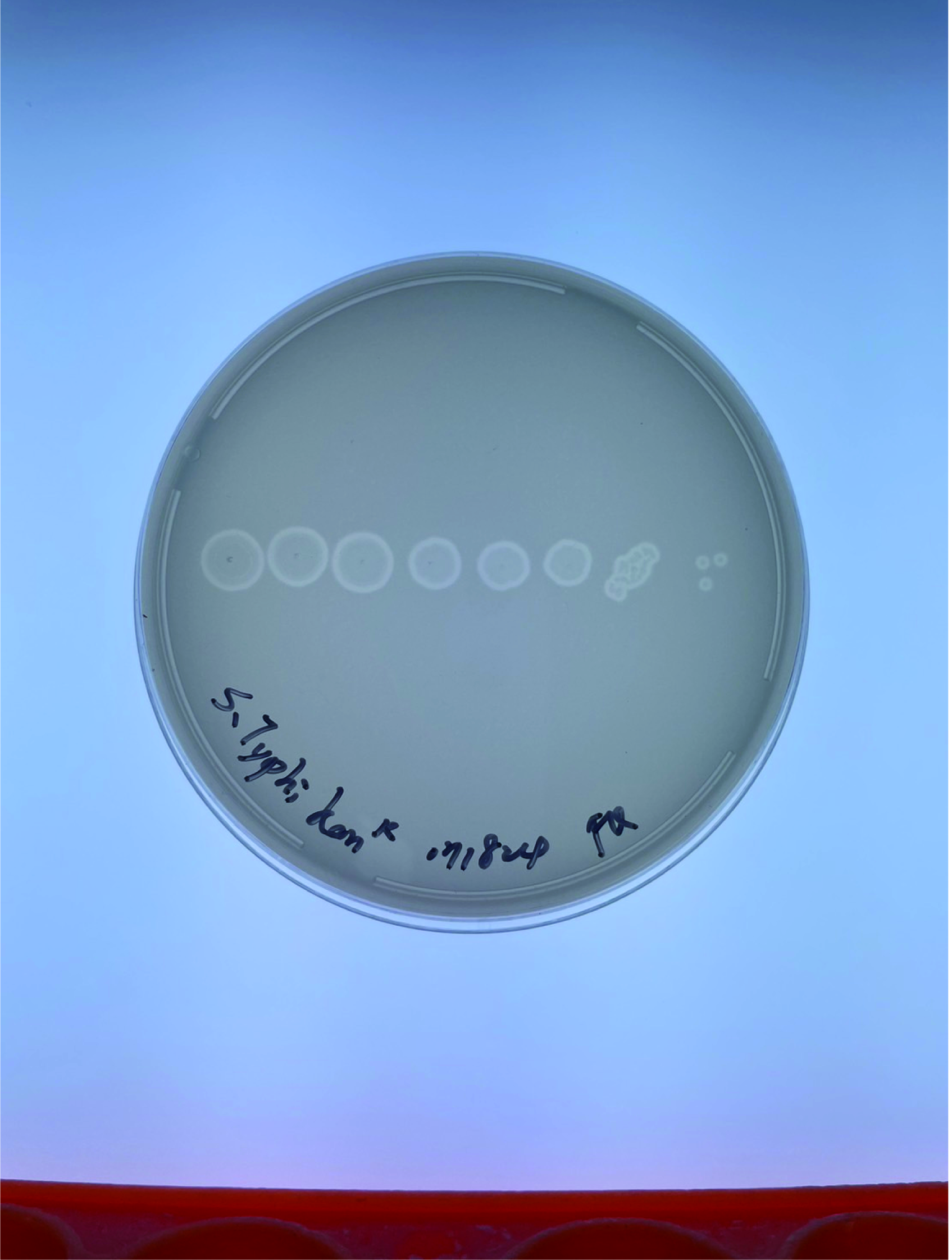

Supplement: Supplementary file 8 — Source data Fig. 4 [file 44318_2025_406_MOESM8_ESM.zip › Source data_Fig. 4/4E/without TSP.tif]

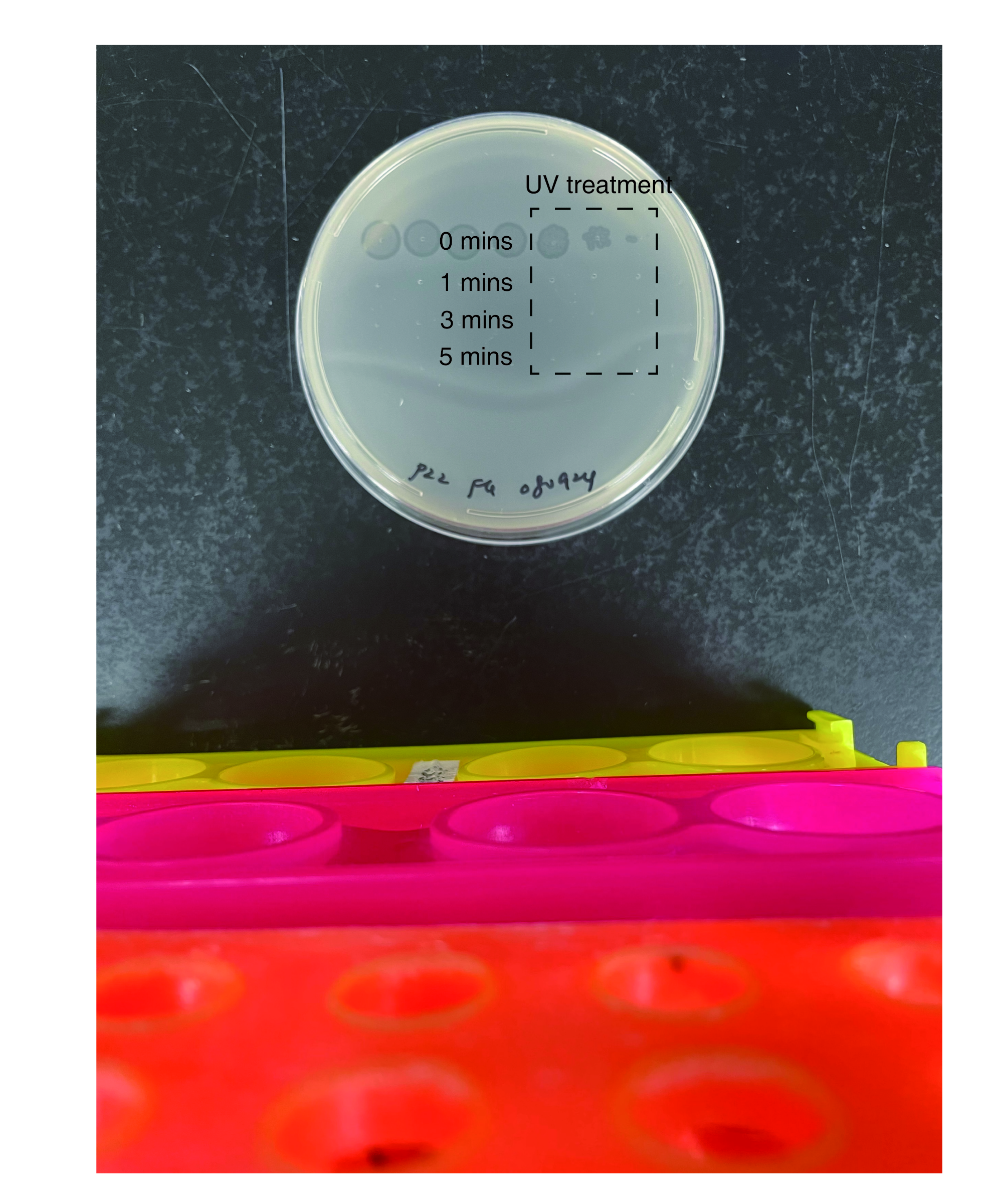

Supplement: Supplementary file 9 — Source data Fig. 5 [file 44318_2025_406_MOESM9_ESM.zip › Source data_Fig. 5/5D/Fig. 5D-spot assay.tif]

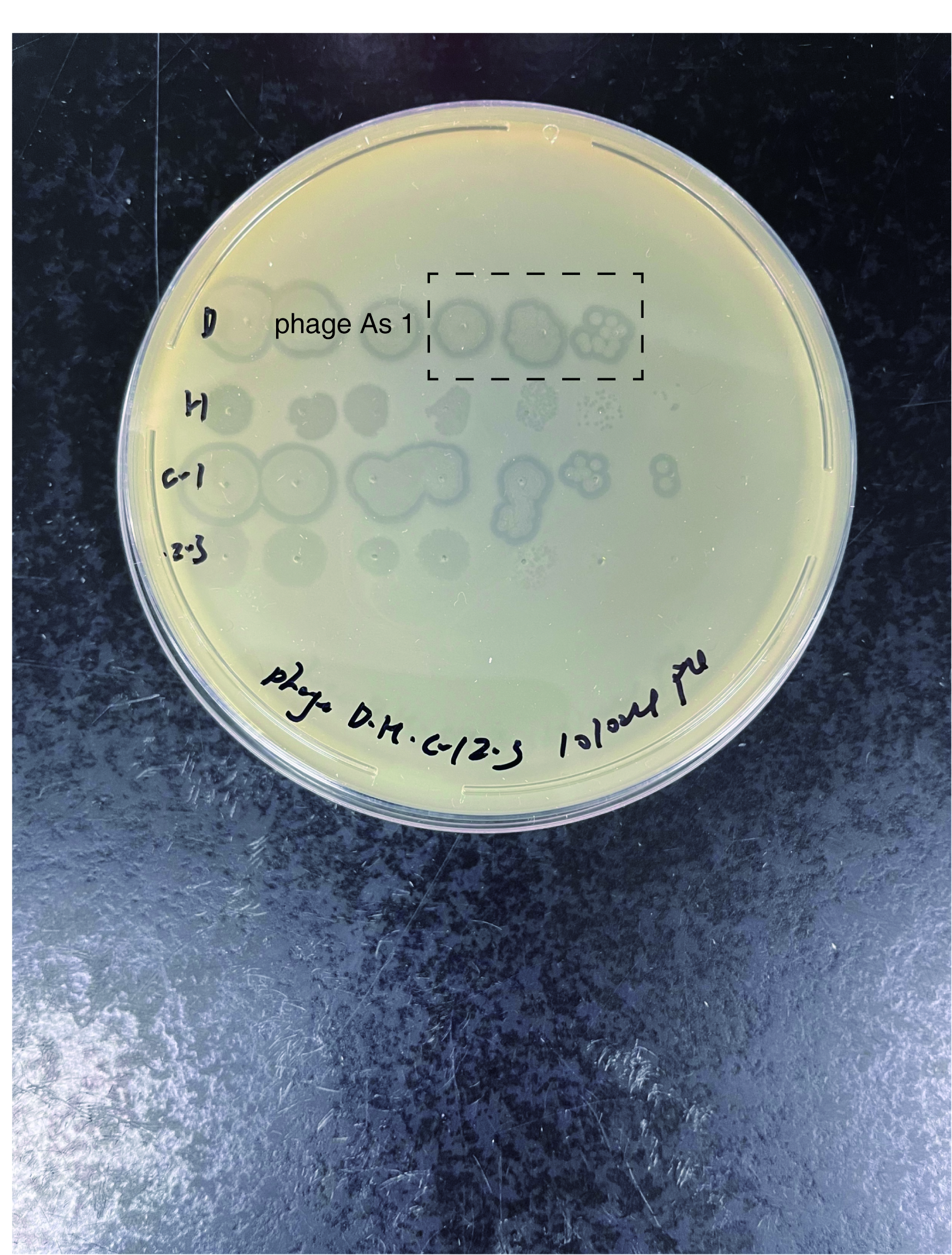

Supplement: Supplementary file 9 — Source data Fig. 5 [file 44318_2025_406_MOESM9_ESM.zip › Source data_Fig. 5/5F/phage As1.tif]

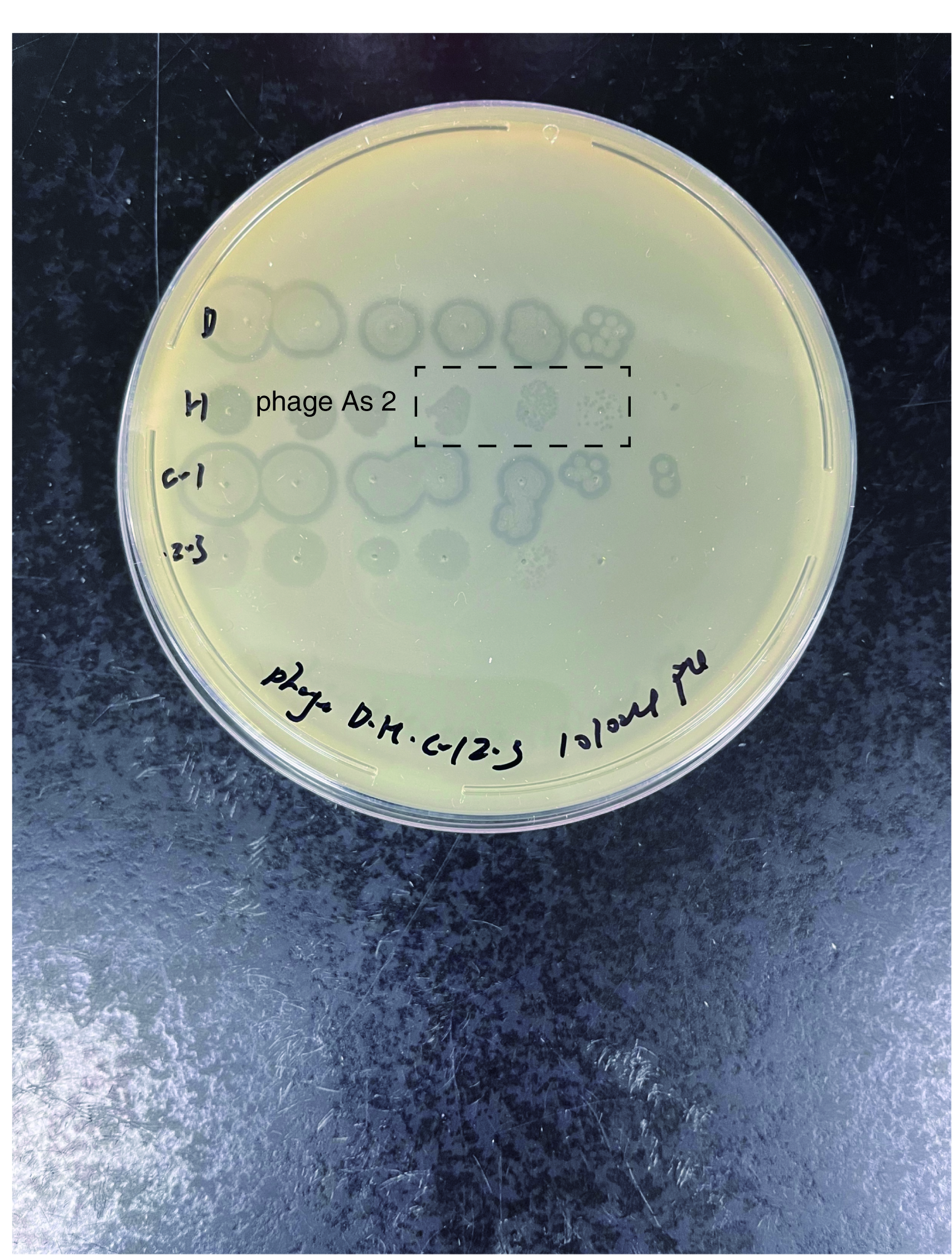

Supplement: Supplementary file 9 — Source data Fig. 5 [file 44318_2025_406_MOESM9_ESM.zip › Source data_Fig. 5/5F/phage As2.tif]

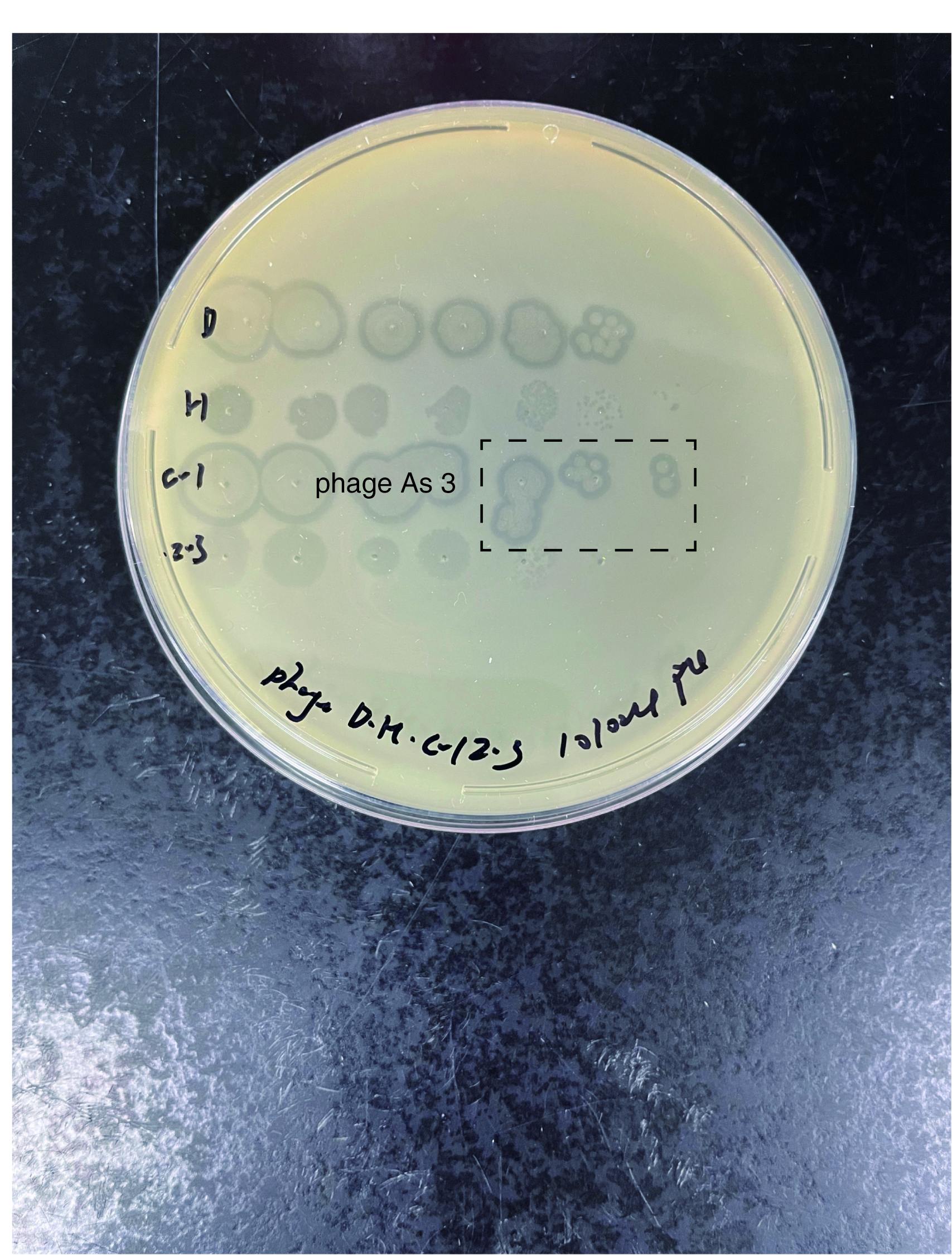

Supplement: Supplementary file 9 — Source data Fig. 5 [file 44318_2025_406_MOESM9_ESM.zip › Source data_Fig. 5/5F/phage As3.tif]

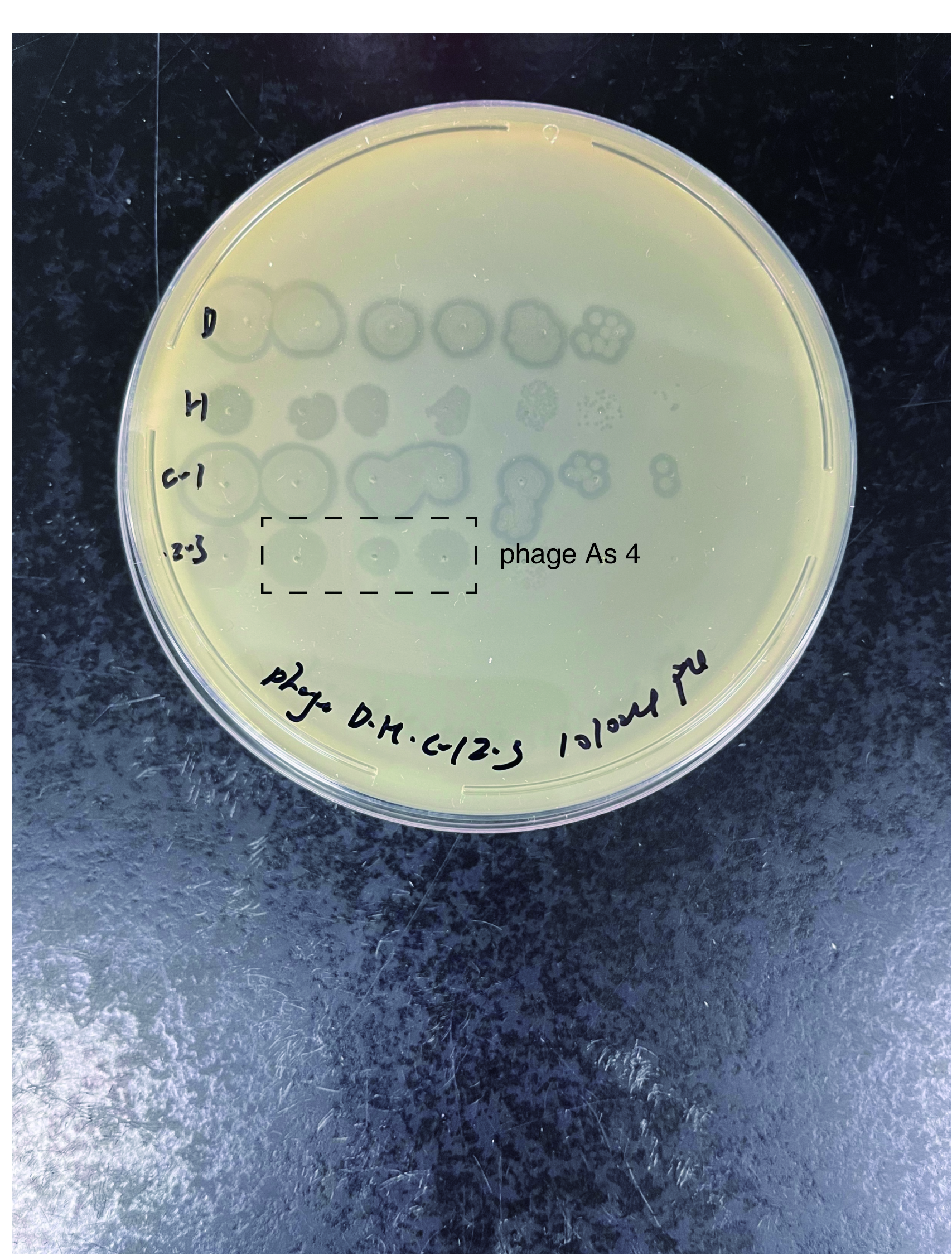

Supplement: Supplementary file 9 — Source data Fig. 5 [file 44318_2025_406_MOESM9_ESM.zip › Source data_Fig. 5/5F/phage As4.tif]

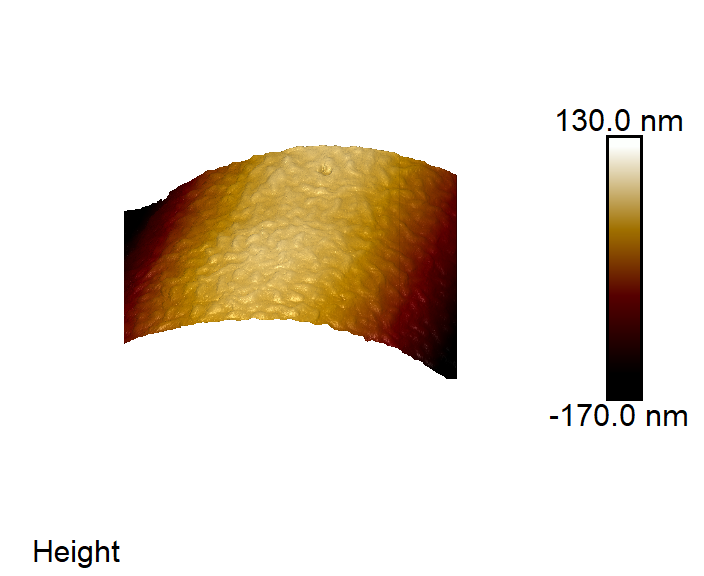

Supplement: Supplementary file 10 — Source data Fig. 6 [file 44318_2025_406_MOESM10_ESM.zip › Source data_Fig. 6/6D/Fig. 6D.tif]

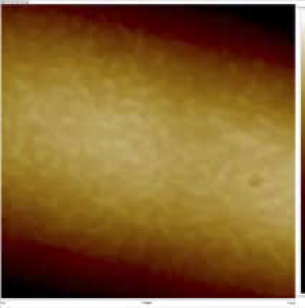

Supplement: Supplementary file 10 — Source data Fig. 6 [file 44318_2025_406_MOESM10_ESM.zip › Source data_Fig. 6/6D/Fig. 6D-raw image in 2D.tiff]

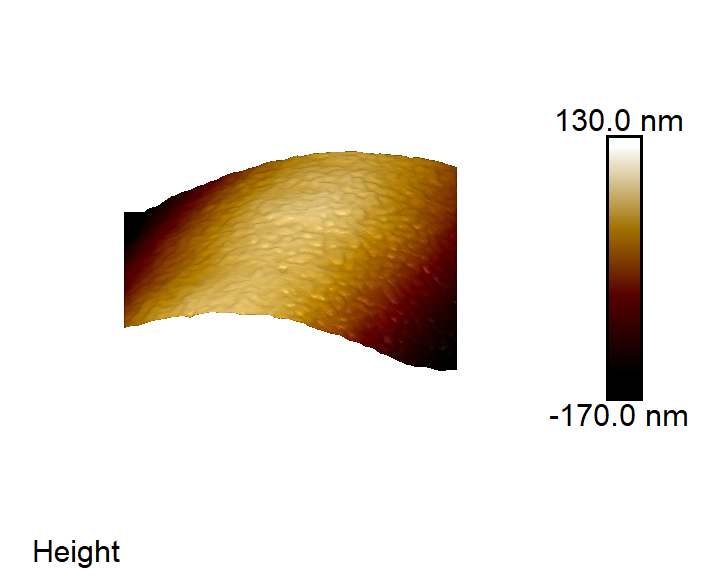

Supplement: Supplementary file 10 — Source data Fig. 6 [file 44318_2025_406_MOESM10_ESM.zip › Source data_Fig. 6/6E/Fig. 6E.tif]

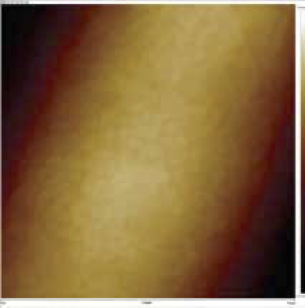

Supplement: Supplementary file 10 — Source data Fig. 6 [file 44318_2025_406_MOESM10_ESM.zip › Source data_Fig. 6/6E/Fig. 6E-raw image in 2D.tiff]

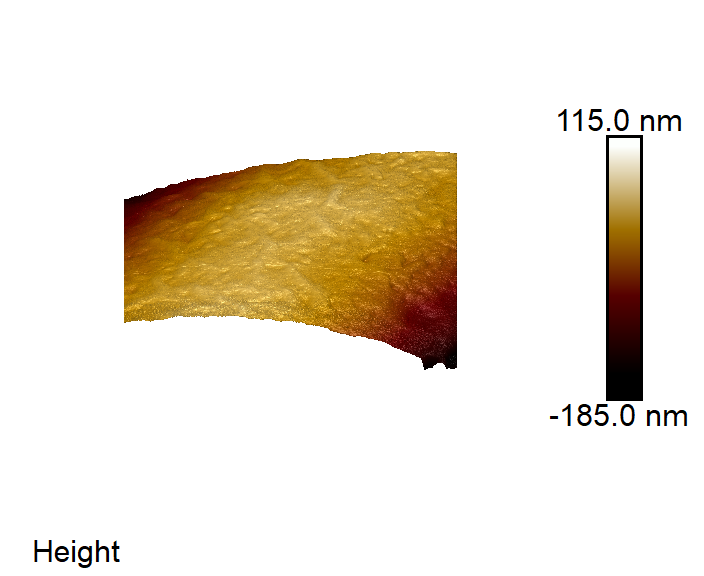

Supplement: Supplementary file 10 — Source data Fig. 6 [file 44318_2025_406_MOESM10_ESM.zip › Source data_Fig. 6/6F/Fig. 6F.tif]

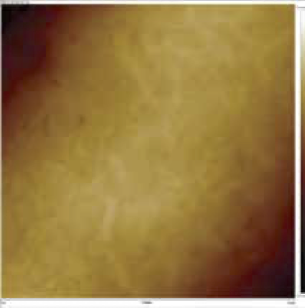

Supplement: Supplementary file 10 — Source data Fig. 6 [file 44318_2025_406_MOESM10_ESM.zip › Source data_Fig. 6/6F/Fig. 6F-raw image in 2D.tiff]
