# Supplementary material for: Surface-mediated bacteriophage defense incurs fitness tradeoffs for interbacterial antagonism
Source: EMBO J. 2025 Mar 10;44(9):2473–500. doi: 10.1038/s44318-025-00406-3 (PMC12048535; doi:10.1038/s44318-025-00406-3)
Supplement: Supplementary file 11 — Expanded View Figures [file 44318_2025_406_MOESM11_ESM.pdf]

Expanded View Figures

Figure EV1. Validation of phage resistance in isolated mutants and monocultural growth experiments.

(**A**) TEM images of model phages used in the study: Felix O1 (left), P22 (middle), and Chi (right). Scale bar = 0.1 µm. (**B**) Tenfold serial dilution spot assay of phages Felix O1 (left), P22 (middle), and Chi (right) on the indicated phage-resistant *S. enterica* strains. The image is representative of triplicate experiments. (**C**) Growth curves of the indicated phage-resistant *S. enterica* strains in LB broth at 37 °C. Wild-type, Δ*waaL*, and Δ*waaG* strains are shown as controls. (**D**) CFUs of the indicated phage-resistant *S. enterica* strains grown at 37 °C for 12 h on LB agar plates. Wild-type, Δ*waaL* (ΔL), and Δ*waaG* (ΔG) strains are shown as controls.

 © The Author(s)

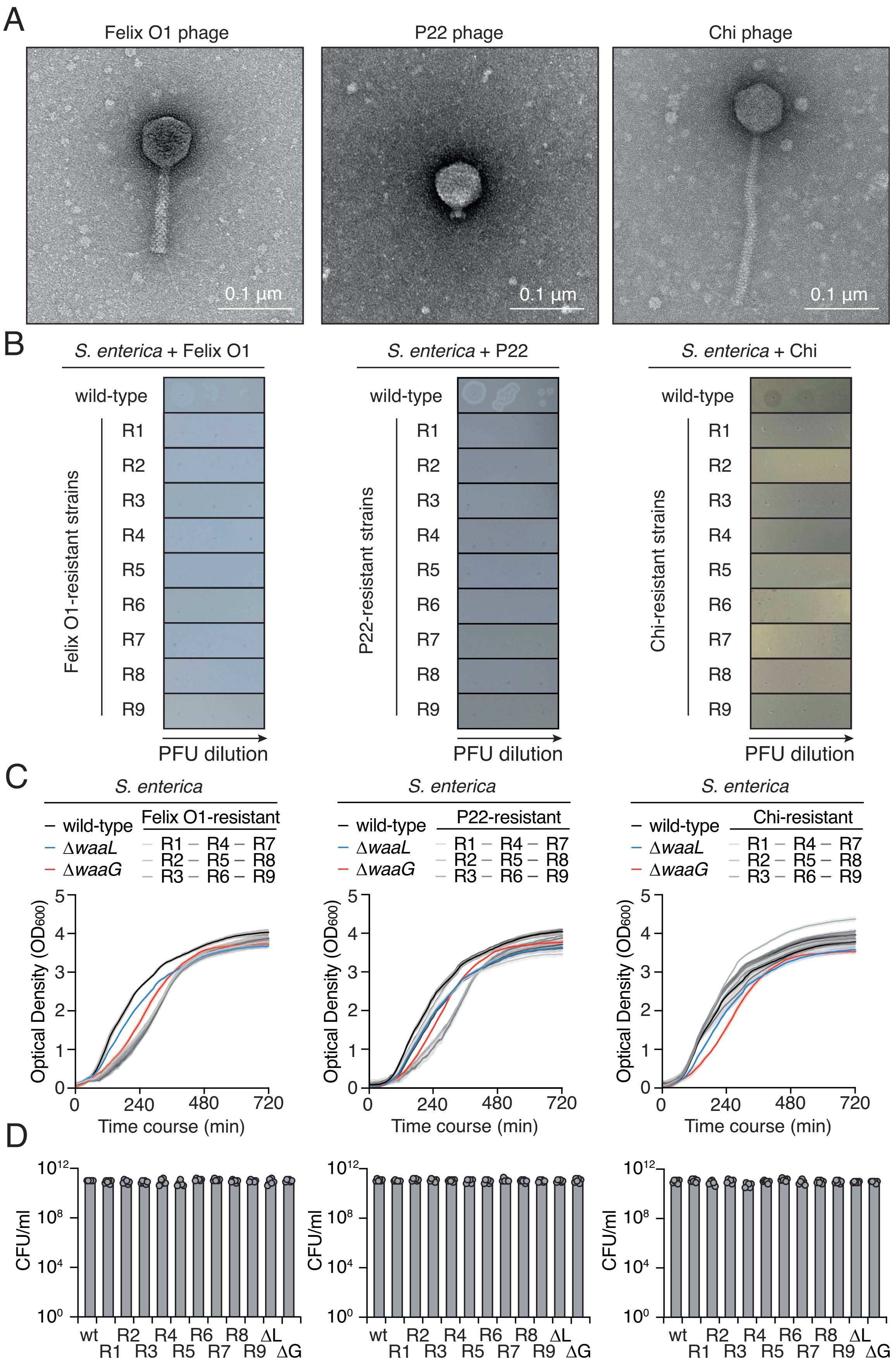
A
Felix O1 phage
P22 phage
Chi phage
0.1 µm
0.1 µm
0.1 µm
B
S. enterica + Felix O1
S. enterica + P22
S. enterica + Chi
wild-type
R1
R2
R3
R4
R5
R6
R7
R8
R9
Felix O1-resistant strains
P22-resistant strains
Chi-resistant strains
PFU dilution
C
S. enterica
wild-type
ΔwaaL
ΔwaaG
Felix O1-resistant
P22-resistant
Chi-resistant
R1 R4 R7
R2 R5 R8
R3 R6 R9
Optical Density (OD600)
Time course (min)
0 240 480 720
D
CFU/ml
10^12
10^8
10^4
10^0
wt R1 R2 R3 R4 R5 R6 R7 R8 R9 ΔL ΔG

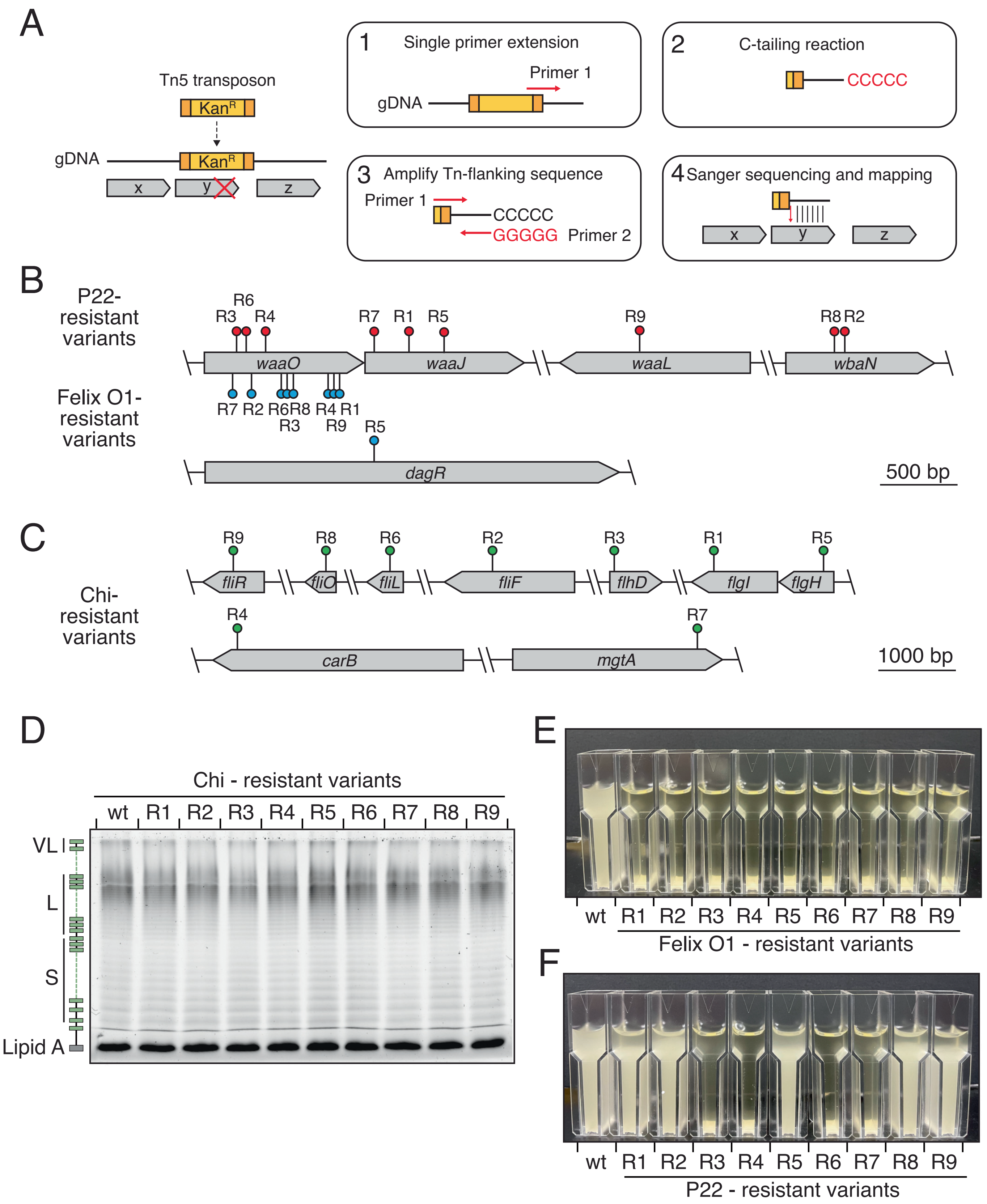
A
Tn5 transposon
KanR
gDNA
KanR
x
y
z
1 Single primer extension
Primer 1
gDNA
2 C-tailing reaction
CCCCC
3 Amplify Tn-flanking sequence
Primer 1
CCCCC
GGGGG Primer 2
4 Sanger sequencing and mapping
x
y
z
B
P22-resistant variants
R6
R3 R4
R7 R1 R5
R9
R8 R2
waaO
waaJ
waaL
wbaN
Felix O1-resistant variants
R7 R2 R6R8 R4 R1
R3 R9
R5
dagR
500 bp
C
R9 R8 R6 R2 R3 R1 R5
fliR
fliO
fliL
fliF
flhD
flgI
flgH
Chi-resistant variants
R4
R7
carB
mgtA
1000 bp
D
Chi - resistant variants
wt R1 R2 R3 R4 R5 R6 R7 R8 R9
VL
L
S
Lipid A
E
wt R1 R2 R3 R4 R5 R6 R7 R8 R9
Felix O1 - resistant variants
F
wt R1 R2 R3 R4 R5 R6 R7 R8 R9
P22 - resistant variants

 © The Author(s)

Figure EV2. Characterization of transposon insertion mutations in phage-resistant *S. enterica* isolates.

(**A**) Schematic workflow for identifying phage-resistant mutations. Detailed procedures are provided in the Methods section. (**B**) Mutated genes in the P22-resistant and Felix O1-resistant *S. enterica* isolates. Scale bar = 500 bp. (**C**) Mutated genes in the Chi-resistant *S. enterica* isolates. Scale bar = 1000 bp. (**D**) 12% LPS PAGE profiles showing LPS levels from the indicated Chi-resistant *S. enterica* strains. A schematic representation of LPS is shown on the left. (**E**, **F**) Macroscopic aggregation analysis to assess LPS deficiency in the indicated Felix O1-resistant (**E**) or P22-resistant (**F**) *S. enterica* strains. The images are representative of triplicate experiments.

© The Author(s)

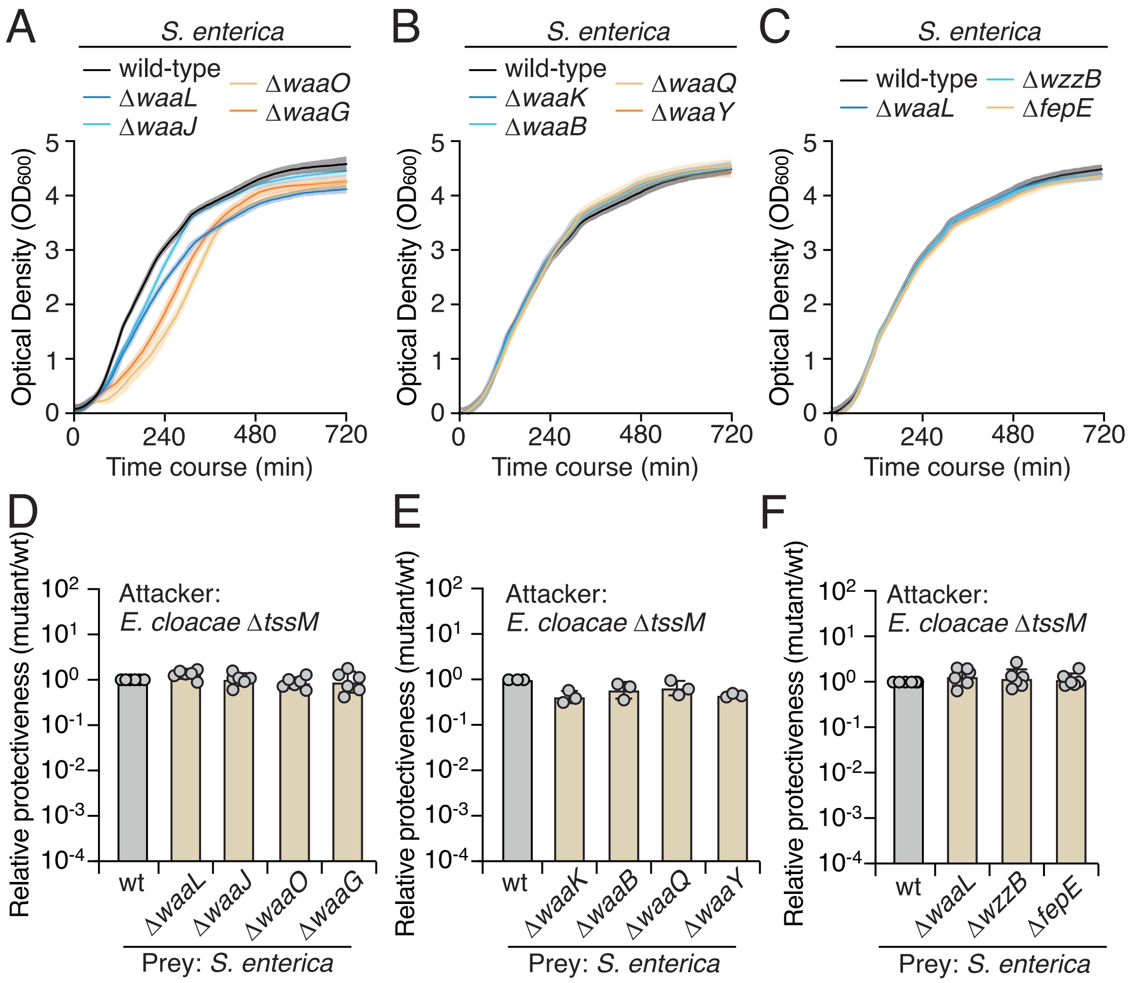

Figure EV3. LPS-deficient *S. enterica* mutants exhibit no significant growth or competition defects.

(**A–C**) Growth curves of the indicated *S. enterica* LPS mutants in LB broth at 37 °C. The wild-type strain is shown as a control. (**D–F**) Protectiveness of the indicated *S. enterica* mutant strains relative to the wild-type strain after competition with *E. cloacae* Δ*tssM*. Data in (**D–F**) are presented as means ± SD ($n = 6$).

© The Author(s)

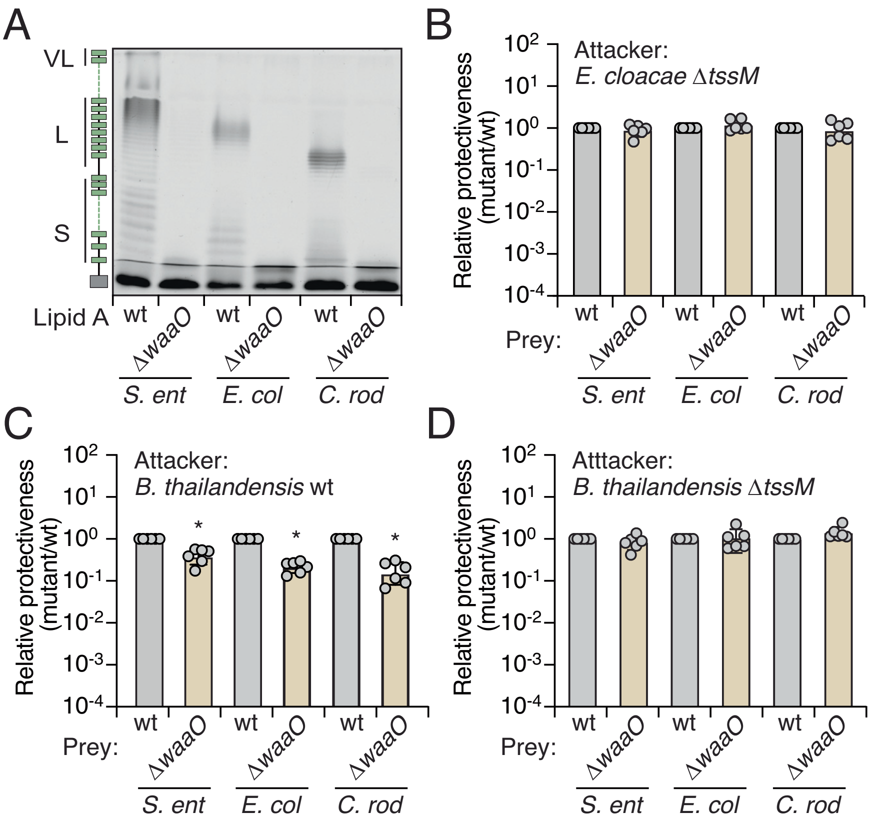

Figure EV4. LPS-deficient bacteria exhibit fitness deficits in competition with antagonistic bacteria carrying active T6SS.

(**A**) 12% LPS PAGE profile displaying LPS levels produced by the indicated *S. enterica*, *E. coli*, and *C. rodentium* strains. (**B**) Protectiveness of the indicated bacterial species (Δ*waaO* mutant) relative to the wild-type strain after competition with *E. cloacae* Δ*tssM*. (**C**, **D**) Protectiveness of the indicated bacterial species (Δ*waaO* mutant) relative to the wild-type strain after competition with *B. thailandensis* wild-type (**C**) or Δ*tssM* (**D**) strains. Data in (**B–D**) are presented as means ± SD ($n = 6$). Asterisks indicate statistically significant differences in the relative protective index between the mutant and wild-type strains ($P < 0.05$).

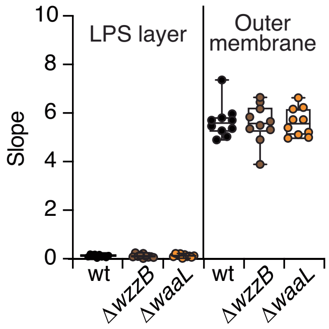

Figure EV5. The slope of AFM force-distance curves.

Two distinct slopes from each force-distance curve were obtained for the *S. enterica* wild-type, Δ*wzzB*, and Δ*waaL* strains. Ten curves were divided into two segments by point 2, as shown in Fig. 6G. The slope measured from the first segment corresponds to the LPS layer (left), whereas the second segment represents the outer membrane (right).

 © The Author(s)
